# Supplementary material for: Eosin Y-Functionalized Upconverting Nanoparticles: Nanophotosensitizers and Deep Tissue Bioimaging Agents for Simultaneous Therapeutic and Diagnostic Applications
Source: Cancers (Basel). 2022 Dec 23;15(1):102. doi: 10.3390/cancers15010102 (PMC9817929; doi:10.3390/cancers15010102)
Supplement: Supplementary file 1 [file cancers-15-00102-s001.zip › cancers-2035025-supplementary.pdf]

Eosin Y-Functionalized Upconverting Nanoparticles: Nanophotosensitizers and Deep Tissue Bioimaging Agents for Simultaneous Therapeutic and Diagnostic Applications

Gabriel López-Peña[1], Silvia Simón-Fuente[2], Dirk H. Ortgies [3, 4, 5], María Ángeles Moliné [6], Emma Martín Rodríguez[1, 4, 5] *, Francisco Sanz-Rodríguez [4, 6] * and Maria Ribagorda[2,5].

[1] Departamento de Física Aplicada, Universidad Autónoma de Madrid, C/ Francisco Tomás y Valiente 7, 28049, Madrid, Spain
[2] Departamento de Química Orgánica, Universidad Autónoma de Madrid, C/ Francisco Tomás y Valiente 7, 28049, Madrid, Spain
[3] Departamento de Física de Materiales, Universidad Autónoma de Madrid, C/ Francisco Tomás y Valiente 7, 28049, Madrid, Spain
[4] Nanomaterials for Bioimaging Group, Instituto Ramón y Cajal de Investigación Sanitaria, Ctra. Colmenar km. 9.300, 28034, Madrid, Spain
[5] Institute for Advanced Research in Chemical Sciences (IAdChem), Universidad Autónoma de Madrid, Spain
[6] Departamento de Biología, Universidad Autónoma de Madrid, C/ Darwin 2, 28049, Madrid, Spain

* Correspondence: emma.martin@uam.es, Tel.: +34 91 497 68 89; francisco.sanz@uam.es, Tel: +34 91 497 82 43.

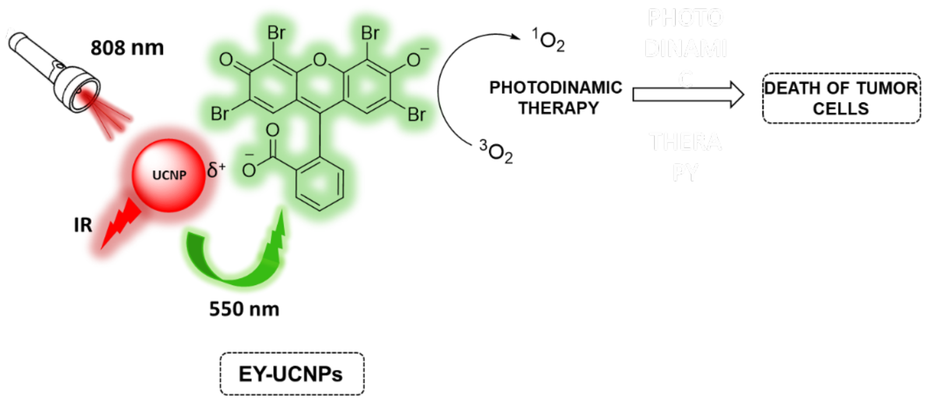

Figure S1. Schematic representation of the working process of the nanoconjugates described in this article, from the initial irradiation of the nanoparticles to the final reactive oxygen species generation.

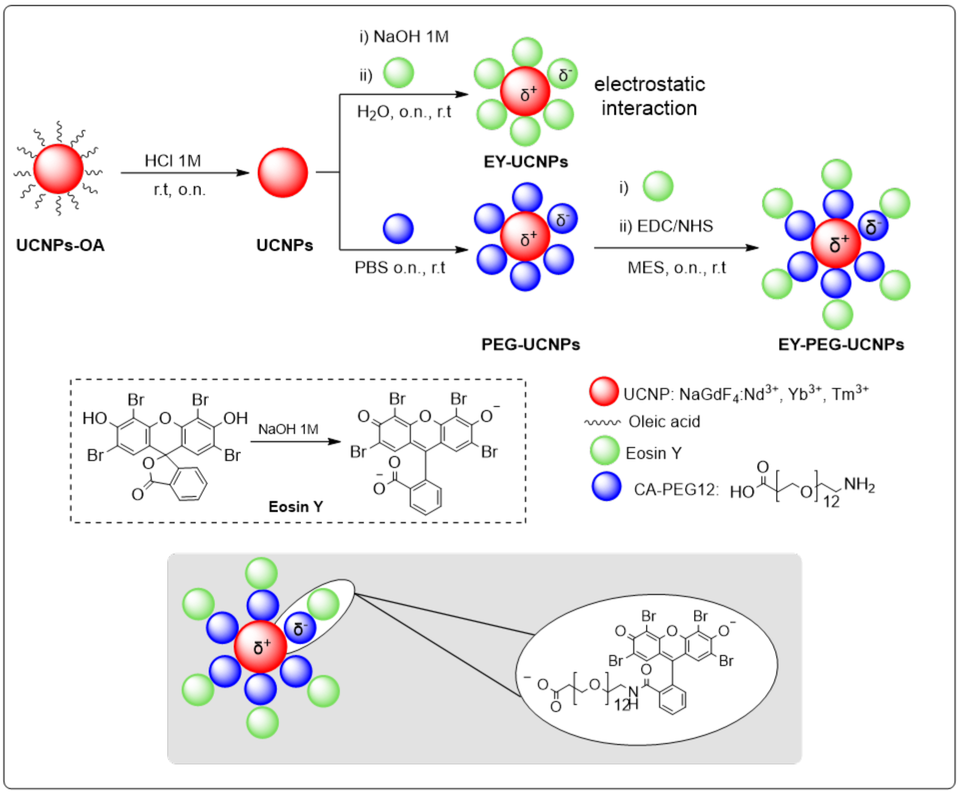

Figure S2. Schematic representation of the different steps involved in the synthesis of the EY-UCNPs and EY-PEG-UCNPs structures.

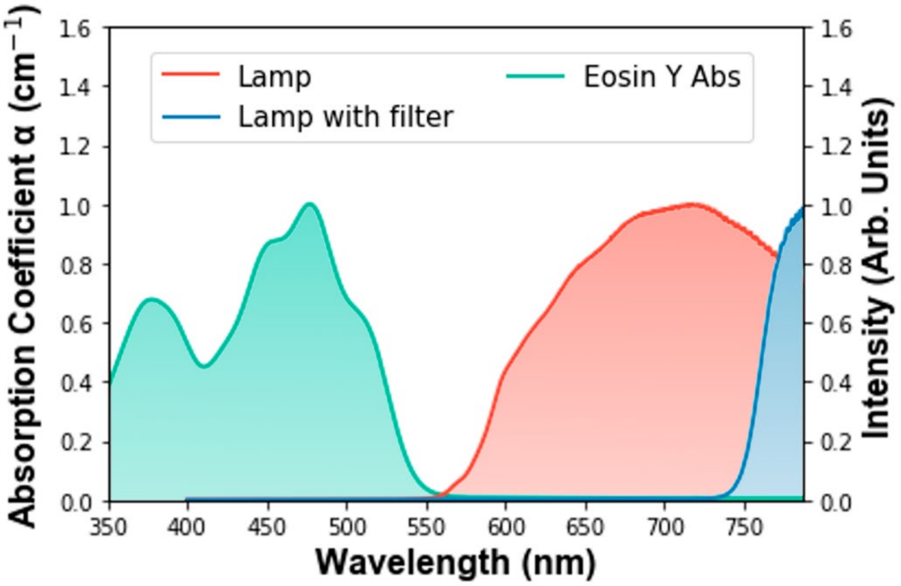

Figure S3. Visible spectra of the lamp used for the irradiation of the cells with (blue) and without (red) a filter to avoid the overlap with eosin Y (green).

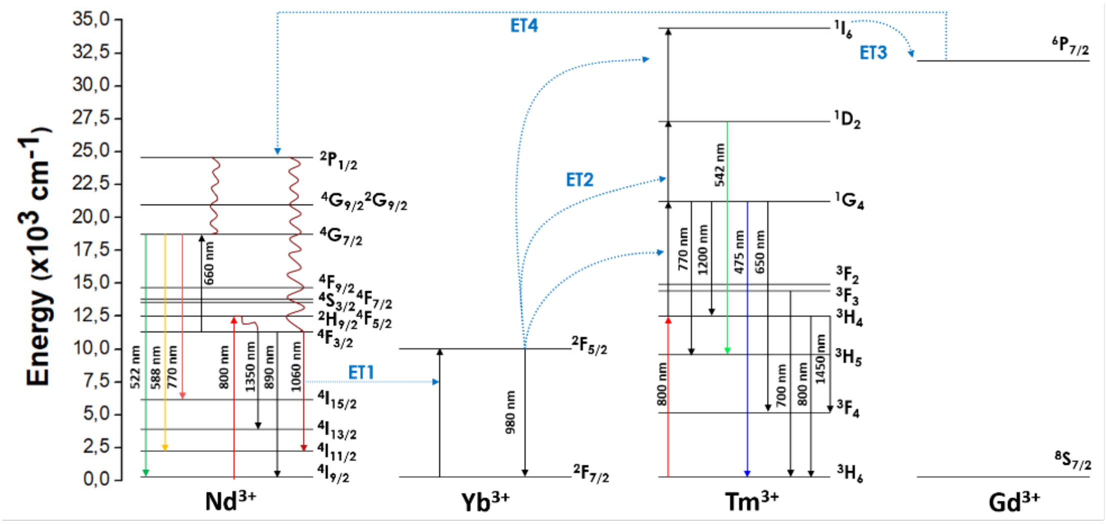

Figure S4. Energy level diagram of the principal ions composing the nanoparticles used in this work. In the diagram both up-conversion and NIR emissions can be observed.

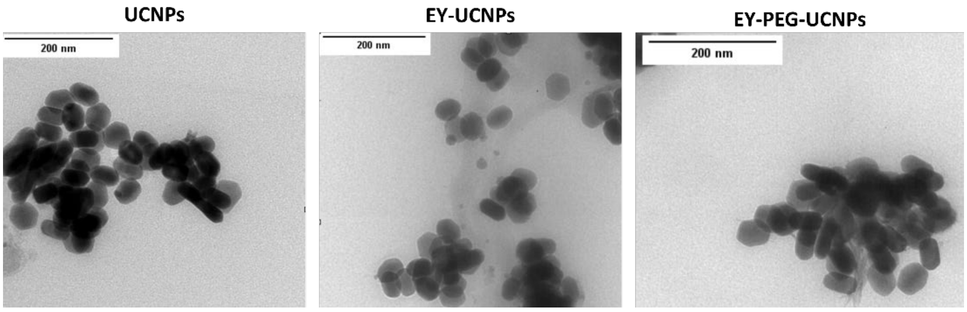

Figure S5. TEM images of UCNPs (left), EY-UCNPs (center) and EY-PEG-UCNPs (right).

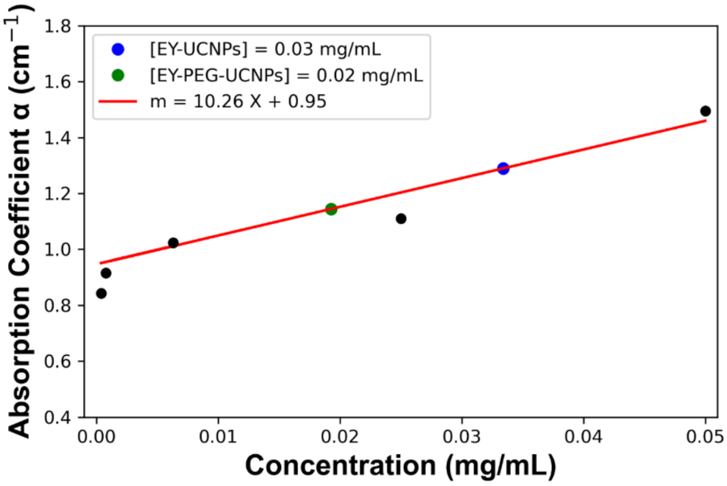

Figure S6. Colorimetric analysis presenting the absorption coefficient for different concentrations of EY. The red line represents the fitting line used for the calculation of the concentration of EY on the surface of the EY-UCNPs and EY-PEG-UCNPs structures (blue and green data points).

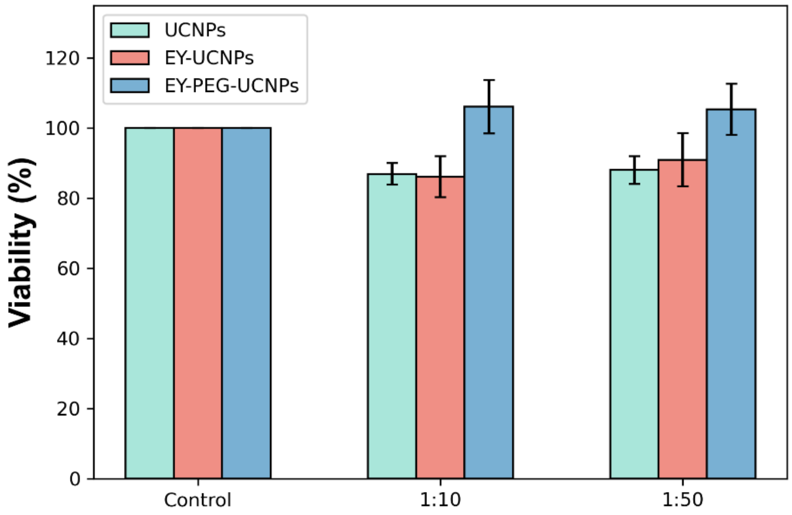

Figure S7. Dark toxicity: Cell survival percentage after incubation with two different concentrations of UCNPs (green), EY-UCNPs (red) and EY-PEG-UCNPs (blue).

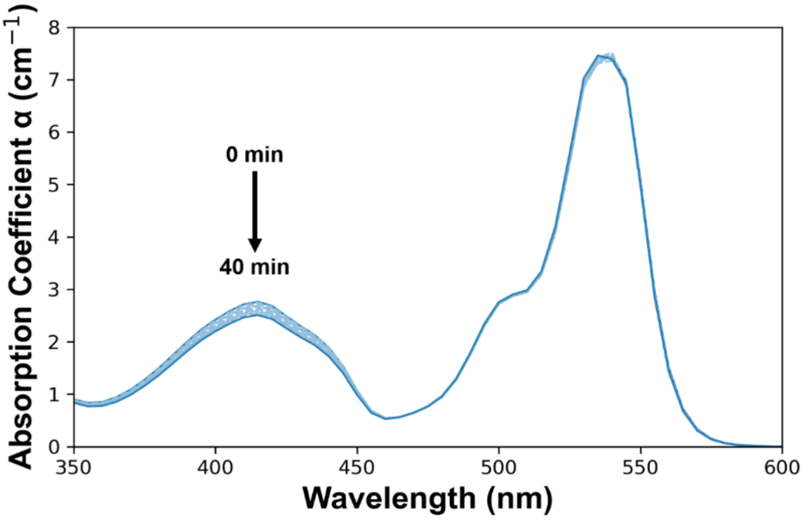

Figure S8. Change in DPBF's absorption band for the EY-PEG-UCNPs sample after irradiating with an 808 nm laser during 40 min.

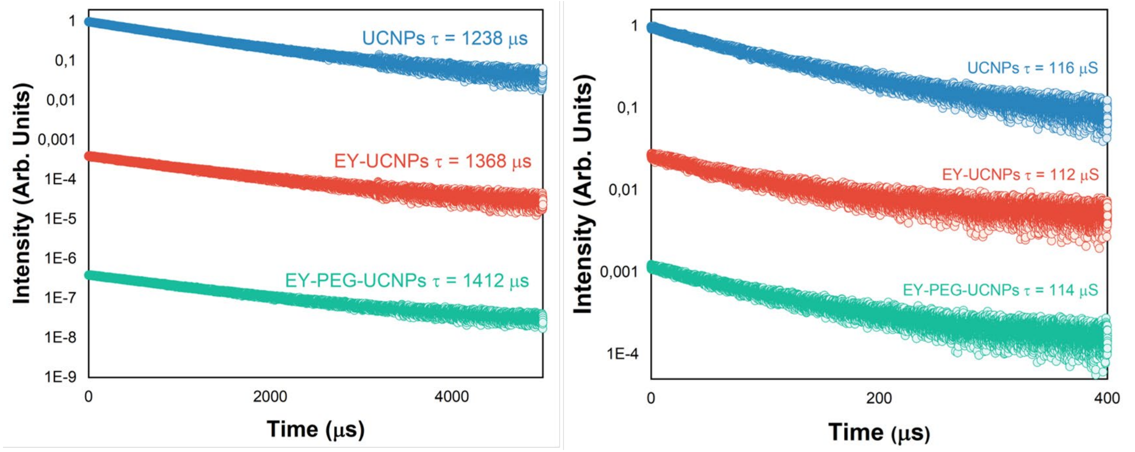

Figure S9. Lifetime comparison of the Nd emission band at 1300 nm (left) and the Yb emission band at 980 nm (right).
